# Cleavage Stage versus Blastocyst Stage Embryo Transfer in Oocyte Donation Cycles

**DOI:** 10.3390/medicina55060293

**Published:** 2019-06-20

**Authors:** George Kontopoulos, Mara Simopoulou, Ioannis Zervomanolakis, Thomas Prokopakis, Kostas Dimitropoulos, Evaggelos Dedoulis, Stylianos Grigorakis, Kristi Agapitou, Eros Nikitos, Anna Rapani, Nikos Vlahos

**Affiliations:** 1 Institute of LIFE Fertility center, IASO Maternity Hospital, 37–39, Kifissias Avenue, 15123 Athens, Attica, Greece; zervomanolakis@womancenter.gr (I.Z.); thomasprokopakis@gmail.com (T.P.); Docdim@otenet.gr (K.D.); dedoulis@gyni.gr (E.D.); siglar@otenet.gr (S.G.); kragapitou@yahoo.gr (K.A.); erosnikitos@hotmail.com (E.N.); 2 Assisted Conception Unit, 2nd Department of Obstetrics and Gynecology, Aretaieion Hospital, Medical School, National and Kapodistrian University of Athens, 76, Vasilisis Sofias Avenue, 11528 Athens, Attica, Greece; gynoffice04@gmail.com; 3 Department of Physiology, Medical School, National and Kapodistrian University of Athens, 75, Mikras Asias, 11527 Athens, Attica, Greece; rapanianna@gmail.com

**Keywords:** cleavage stage, blastocyst stage, embryo transfer, frozen-thaw oocyte, donation cycle

## Abstract

*Background and Objective:* During the last few years, a trend has been noted towards embryos being transferred at the blastocyst stage, which has been associated with improved rates regarding implantation and clinical pregnancy in comparison to cleavage stage embryo transfers. There is a limited number of studies investigating this notion in oocyte donation cycles employing cryopreserved embryos. The aim of this study is to evaluate the implantation potential and clinical pregnancy rates between the day 3 cleavage stage and blastocyst stage embryo transfers in oocyte donation cycles employing vitrified embryos. *Methods:* This is a retrospective evaluation of oocyte donation frozen–thawed transfers completed in our clinic from January 2017 to December 2017. Intracytoplasmic sperm injection was conducted for all oocytes. Following fertilization, all embryos were cryopreserved either at the cleavage or blastocyst stage. Embryo transfer of two embryos was performed under direct sonographic guidance in all cases. *Results:* Our results confirmed a 55.6% clinical pregnancy (CP) resulting from day 3 embryo transfers, a 68.8% CP from day 5, and 71.4% CP from day 6. Significantly improved pregnancy rates were related to embryo transfers at the blastocyst stage when compared to cleavage stage transfers (68.9% and 55.6% respectively, *p* = 0.016). The risk with regards to multiple pregnancies was similar. *Conclusion:* Our findings indicate that in oocyte donation cycles employing vitrified embryos, embryo transfer at the blastocyst stage is accompanied with a significant improvement in pregnancy rates and merits further investigation.

## 1. Introduction

A remarkable increase in the utilization of oocyte donation has been noted since an array of infertility issues may be effectively addressed and managed employing this option. Age related infertility, recurrent implantation failures, diminished ovarian reserve, poor response to ovarian stimulation, recurrent miscarriages [1], as well as genetically associated dysfunctions fuel the need for oocyte donation. Despite reports that oocyte donation may be associated with a higher risk of complications, such as preeclampsia, low birth weight and preterm birth, along with poor neonatal outcomes [2,3,4], it often constitutes the sole and last available option for certain patients to achieve pregnancy.

Transfer of embryos at the blastocyst stage has surfaced as a prominent approach for the majority of in vitro fertilization (IVF) units. Several reports support that in fresh IVF embryo transfer (ET) cycles, blastocyst transfer is associated with improved live birth rates in comparison to early cleavage stage embryo transfers [5,6], while others still debate whether embryo transfer at the blastocyst stage is superior to day 3 [7,8]. There is even less data regarding the optimal time of embryo transfer when referring to oocyte donation cycles and, furthermore, regarding oocyte donation frozen-thawed transfers. The optimal developmental stage of the embryo merits further investigation [9]. In regards to the number of embryos transferred, it should be stated that a restricting policy is well established in various countries based on the patient’s age as well as the number of previous cycles performed. In cases of patients receiving donor eggs, the legislation in Greece dictates the practitioners may proceed employing the maximum number of two embryos with patient desire identified as a major factor in the decision-making process. The phenomenon of multiple pregnancies constitutes one of the most crucial complications associated with IVF [1]. The detrimental consequence of multiple pregnancies has been acknowledged, and documented as adding another level of complexity, thus a good practice recommendation is commonly in place by several governments. The sole way to ensure prevention of multiple pregnancies is the elective single embryo transfer (eSET) approach, with randomized clinical trials (RCTs) supporting that when good quality embryos are available, eSET does not decrease the success rate [2].

Vitrification is the cryopreservation technique of choice which has taken the IVF world by storm [10], providing impressive results, good quality cleavage and blastocyst development rates, extending to live birth rates and encouraging obstetric and perinatal results [11,12,13]. However, the unpredictable consequences attributed to the prolonged storage period oocytes and embryos are exposed to are yet to be conclusively elaborated [14].

The purpose of this study is to analyze the impact of transferring cryopreserved thawed embryos obtained from oocyte donors in different developmental stages in order to identify the best approach for these patients.

## 2. Materials and Methods

This is a retrospective evaluation of 303 embryo transfers following oocyte donation performed in our center during the period from January 2017 to December 2017. Ovarian stimulation of the donors was performed according to a short, step down Gonadotropin-releasing hormone (GnRH) antagonist stimulation protocol with a recombinant Follicle-stimulating hormone (FSH) (folitropin alpha) (Gonal-F, (Merck Serono Europe Ltd., London, UK) 250 IU/day) starting on the 3rd day of the menstrual cycle. All patients were re-evaluated on day 5 of stimulation with a trans-vaginal ultrasound examination and serum estradiol levels. At this time, adjustments to the dose were made and a GnRH antagonist (Cetrotide, (Merck Serono Europe Ltd., London, UK) 0.25 mg/day) was added to the regimen. Triggering was performed either with a recombinant human Chorionic Gonadotropin (hCG) (Ovitrelle, (Merck Serono Europe Ltd., London, UK) 250 μg, SQ) or with a GnRH agonist (Triptorelin, 0.2 mg, SQ IPSEN EPE, Athens, Greece) when peak estradiol levels exceeded 2500 pg/mL. All donor oocytes were cultured in Life Global Total single media (7% CO_2_, 5% O_2_, approx. 7.28 pH), as well as employing dry culture conditions. Intracytoplasmic sperm injection (ICSI) was performed for all donor oocytes. Following the fertilization stage, all embryos were vitrified either at the cleavage or blastocyst stage employing the Kitazato/Cryotop method (DMSO/EG) according to Kuwayama. The degeneration rate recorded was 1.2%. Grading of the cleavage stage embryos was performed according to Veeck [15], while blastocyst stage embryos were graded according to Gardner et al. [16]. The term “good quality embryo” was engaged in order to describe embryos of high grade namely 7–8 cells stage of grade 1 and 2 at the cleavage stage, and 4 AA, 4 AB, 5 AA, 5 AB, 6 AA, and 6 AB at the blastocyst stage. Endometrial preparation of the recipients was performed according to a standard down regulation protocol with a GnRH agonist for at least 4 weeks in order to avoid random follicular development. Sonographic evaluation and a baseline hormonal profile to confirm down regulation of the HPO (Hypothalamic-Pituitary-Ovarian) axis were performed for all recipients. Endometrial stimulation was initiated with estradiol patches (Dermestril (Rottapharm Hellas A.E., Athens, Greece), 100 MCG/24 h) every other day for at least 14 days. The patients were evaluated on a weekly basis for endometrial morphology and thickness. Providing that a tri-laminar endometrium with a thickness of at least 8 mm was confirmed, additional progesterone supplementation in the form of vaginal micronized progesterone 200 mg (Angellini Pharma Hellas A.B.E.E., Athens, Greece) was initiated starting on the night of the 4th day prior to the scheduled transfer for day 3 thawed transfers, or the night of the 6th day for thawed blastocyst transfers. Embryo transfer was performed under direct sonographic guidance with a soft pass embryo transfer catheter (Cook Medical, Limerick, Ireland) or a Wallace Sure view (Cooper surgical, Life Science ChemiLab S.A., Athens, Greece). In case the transfer was not successful with these soft catheters, a Wallace malleable stylet was used. In all cases, 2 embryos were transferred. Luteal support continued as described for an additional 12 days when a blood pregnancy test was performed. A transvaginal ultrasound was performed two weeks later to confirm an intrauterine pregnancy with a positive fetal heart rate. For ongoing pregnancies, luteal phase support continued up to the 9th week of gestation.

Statistical analysis was performed employing a commercially available statistical program (SPSS, v25, Chicago, IL, USA). For comparisons between proportions, the Pearson Chi square test was performed. The data distribution was evaluated employing the Shapiro–Wilk test. Since all data was normally distributed, *t* test was employed. A p value of less than 0.05 was considered significant. The study was approved by the ethics committee of the IASO maternity hospital and the scientific board of the IOLIFE fertility center (IRB: Approval number: 37/25-05-2019; date of project approval: 08/03/2018).

## 3. Results

Three hundred and three (303) frozen–thaw transfers from 301 oocyte donation cycles were included in the study. One hundred seventy-one (171) day 3 transfers were performed resulting in 95 clinical pregnancies (55.6%). One hundred and twenty-five (125) day 5 embryo transfers were also performed resulting in 86 clinical pregnancies (68.8%). Seven (7) day 6 embryo transfers were included in this study, reporting 5 clinical pregnancies (71.4%). No difference was recorded in terms of the donors’ age, day-2 FSH, antral follicle count, number of MII oocytes and number of good quality embryos between the two groups (Table 1). Regarding the type of the catheter employed and the documented difficulty during the transfer procedure in the two groups, no difference was reported. A comparison between embryo transfer on day 3 and the blastocyst stage was performed with regards to the rates of clinical and ongoing pregnancy. Embryo transfers on day 5 or day 6 were included in the same group and consequently commonly analyzed. To conclude, a significant association between blastocyst stage transfers and enhanced pregnancy rates was observed, since 91 clinical pregnancies were reported out of 132 transfers (68.9%) in comparison to cleavage rate transfers where 95 clinical pregnancies were reported out of 171 transfers (55.6%, *p* = 0.016). The risk of multiple pregnancies (mainly twins) was similar in the two groups (12 out of 91 pregnancies (13.18%) in the blastocyst group vs 14 out of 95 (14.73%) in the day-3 transfers (*p* = 0.9559).

## 4. Discussion

Our findings suggest that in oocyte donation cycles where all embryos are cryopreserved, a thawed embryo transfer at the blastocyst stage is associated with a significant improvement in pregnancy rates. The age of the oocyte donor constitutes an element of paramount importance for the pregnancy success and should be meticulously acknowledged prior to initiating a donor cycle [17]. The fact that the mean number of the donor’s age in both cleavage and blastocyst transfer in our study was approximately 24 years old, enabled a valid analysis and safely ascertained further comparisons on outcome measures.

The day-3 versus day-5 embryo transfer debate has been an area of scientific interest for the last two decades. Numerous studies and scientific groups have published their results and thorough observations regarding the optimal time of embryo transfer [18]. Certain advantages and disadvantages are involved in the decision of day 3 versus day 5 embryo transfer in fresh cycles [19]. The increased risk of developmental arrest and the risk of epigenetic changes due to the prolonged culture should always be considered when contemplating day-3 versus day-5 embryo transfer. However, certain established facts serving as advantages should be taken into consideration, such as the safety of the additional time provided ensuring proper embryo assessment [20].

Following years of research, collective data can now provide evidence supporting the day-5 transfer [21,22]. Nevertheless, there are several issues that may interfere with the decision for autologous blastocyst transfer, especially the risk of ending with no embryos available for transfer on day 5. In fact, the decision for blastocyst stage embryo culture may have been over idealized in literature due to the preselection of the patient population. Studies have shown that in the era of personalized medicine the decision to extended culture should be in relation to not only the age of the patient, but also the functionality of ovarian response being low or normal [23]. In practice, blastocyst stage embryo transfer when unjustifiably employed for poor prognosis patients may in fact lead to failure to reach the embryo transfer stage and conclude the cycle. Such faulty practice, may in turn decrease significant clinical pregnancy and live birth rates, and therefore, should be considered contraindicated [24]. On the same concept, studies show that criteria for blastocyst transfer may vary from institution to institution and may involve laboratory quality issues such as the fact that digital analysis may be employed to quantitatively evaluate blastocysts versus plain microscopy evaluation [25]. Furthermore, meta-analysis studies show that medium selection being single step or sequential still merits investigation regarding the optimal approach to blastocyst culture [26]. Stimulation performance consists of another parameter well-argued in literature that may be strongly associated with blastocyst development. A study by Braga et al., 2012, shows that the FSH dosage and the number of retrieved oocytes may adversely impact blastocyst quality [27]. Milder ovarian stimulation protocols have been proposed to reduce the possibility of segregation errors during preimplantation development [28].

Whereas there is a plethora of evidence regarding the day of transfer in fresh autologous transfers, there is moderately scarce information regarding oocyte donation cycles rendering a possible comparison to our data of rather limited significance. Furthermore, little is known regarding the optimal time of transfer in frozen thawed oocyte donation cycles. In agreement with our results regarding the clinical pregnancy rate, Schoolcraft and Gardner (2000) [29] highlighted the superiority of blastocyst transfer on donor cycles with a 65.8% implantation rate and a 87.6% pregnancy rate, while cleavage stage transfers resulted in 47.1% and 75% respectively. Moreover, a significantly improved clinical pregnancy rate for the blastocyst transfer in oocyte donation cycles compared to cleavage stage (73% vs 40%) has been indicated in a recent study [30]. Nonetheless, it should be underlined that blastocyst stage transfer was performed on day 6 according to authors, hence any comparison of this data to our results, should be attempted with caution. In regards to autologous frozen cycles, numerous studies in literature have attempted to address the debate on cleavage versus blastocyst stage embryo transfers. A system study including 236,191 cycles, reported observations of 49% increased odds of live birth rates, 68% increased odds of clinical pregnancy, and 7% decreased odds of miscarriage following blastocyst stage FET when compared to cleavage stage FET. However, it should be further highlighted that the 16% increased odds of preterm delivery in blastocyst stage FET [31] that was documented, may raise hesitation in regards to accepting blastocyst stage transfer as the optimal day of transfer. Furthermore, opting for a blastocyst stage ET may not be the superior choice in light of the fact that the perinatal outcomes in either days of transfer are presented with no difference [31]. As anticipated, this heated debate is supported by opposite schools of thought that are both based on solid data, portraying the undoubtable heterogeneity attributed to the different practitioners and clinics. Thus, it has been voiced that the stage of freezing may not exert an impact on the implantation success rate or the clinical pregnancy rate. On a recent meta-analysis, the clinical pregnancy rate between vitrification warming cycles on cleavage or blastocyst stage poses no difference (Risk Ratio 0.97, 95% Confidence Interval) despite the fact that the implantation rate was higher for the blastocyst transfers [32].

Furthermore, significantly higher pregnancy rates have been reported in cases of frozen–thawed embryo transfer cycles in comparison to fresh cycles [33,34]. This is thought to be attributed to the supra-physiologic steroid levels during the stimulation that may adversely affect the endometrial receptivity [35]. In contrast, cryopreserved cycles permit better synchronization between the recipient’s endometrium and the embryo(s) transferred.

In has been a standard practice in our center, mainly due to logistic reasons, to employ vitrification in order to cryopreserve all embryos obtained from oocyte donation cycles. In addition, there is a restricted policy in our center not to transfer more than two embryos. The decision to proceed with ET on day 3 or day 5 was based on the patients’ will, following informed consent. All patients were informed of the probability of cancellation of ET following blastocyst culture which has been reported to be at 2.6% based on a meta-analysis study by Glujovsky et al., 2016 [7]. With respect to the criteria employed in safely subjecting embryos to blastocyst culture, there are numerous studies reporting data. In our clinic, the patient selection criteria agree with the publication by Thum et al., 2010 [36]. Particularly patients below the age of 38 were selected for blastocyst culture when they were presented with at least four retrieved oocytes, four zygotes, and at least two 8-cell and 2 6/7-cell embryos of top quality.

Based on the data collected in our study, blastocyst stage transfer is associated with higher pregnancy rates as compared to cleavage stage transfers (68.9% vs. 55.6%). The risk regarding multiple gestations seems to be similar between the two approaches, which is contracting evidence in regards to published literature. Blastocyst embryo transfer is believed to minimize the risk of multiple gestations [37].

## 5. Conclusions

Our findings suggest that oocyte donation cycles employing frozen thawed embryo transfer at the blastocyst stage are associated with an improvement in pregnancy rates. Further evaluation concluding on other aspects, such as the perinatal and obstetric outcome related to this approach should be thoroughly assessed prior to concurring on the ideal developmental stage for embryo transfer in oocyte donation cycles. Patients embarking on oocyte donation present a unique population in terms that they represent a good prognosis group, characterized by homogeneity when compared to infertile patients proceeding with autologous IVF treatment. Further data on the optimal frozen–thawed embryo transfer stage in oocyte donation cycles could certainly buttress current findings and help shape the clinical practice.

## Figures and Tables

**Table 1:** Data regarding embryo transfers from oocyte donation cycles, employing cryopreserved embryos.

	Cleavage Stage ET		Blastocyst ET		*p*-Values
	Mean ± SD	Range	Mean ± SD	Range	
**Donor Age (years)**	24.74 ± 3.58	18−34	24.68 ± 3.61	18−34	0.88
**AMH Levels (ng/mL)**	4.9 ± 1.06	2.59−7.32	4.74 ± 1.04	2.57−7.22	0.20
**FSH Levels (mlU/mL)**	6.62 ± 1.57	4.1−9.2	6.83 ± 1.68	4.0−9.0	0.27
**Antral Follicle Count**	8.37 ± 1.85	4−12	8.11 ± 2.11	4−12	0.26
**Number of MII Oocytes Retrieved**	7.49 ± 1.89	3−11	7.21 ± 1.73	3−11	0.17
**Recipient Age (years)**	42.51 ± 4.14	31−49	42.72 ± 3.97	38−50	0.65
**Number of Day 3 Top Quality Embryos**	5.43 ± 0.77	3−7	5.3 ± 0.73	4−8	0.13

All the above parameters were normally distributed; a *t* test was employed.
